# Diagnostic and Clinical Impact of Double-Balloon Enteroscopy in Small-Bowel Inflammatory Lesions: A Retrospective Cohort Study in a Turkish Population

**DOI:** 10.3390/diagnostics15060661

**Published:** 2025-03-09

**Authors:** Suleyman Dolu, Mehmet Emin Arayici, Soner Onem, Huseyin Dongelli, Mesut Akarsu

**Affiliations:** 1 Department of Gastroenterology, Faculty of Medicine, Dokuz Eylul University, 15 July Medicine and Art Campus, Inciralti-Balcova, Izmir 35340, Turkey; 2 Department of Biostatistics and Medical Informatics, Faculty of Medicine, Dokuz Eylul University, Izmir 35340, Turkey; mehmet.e.arayici@gmail.com; 3 Department of Public Health, Faculty of Medicine, Dokuz Eylul University, Izmir 35340, Turkey; 4 Department of Gastroenterology, Samsun Training and Research Hospital, Samsun 55090, Turkey; 5 Department of Internal Medicine, Faculty of Medicine, Dokuz Eylul University, Izmir 35340, Turkey; 6 Private Gastroenterology Clinic, Izmir 35000, Turkey

**Keywords:** inflammatory lesions, small bowel, diagnostics, double-balloon enteroscopy

## Abstract

**Background/Objectives:** Small-bowel inflammatory lesions are challenging to diagnose thanks to their anatomical complexity and the limitations of conventional imaging. Double-balloon enteroscopy (DBE) allows for direct visualization, biopsy, and therapeutic intervention. This study evaluated the diagnostic yield and clinical impact of DBE in small-bowel inflammatory lesions, particularly in differentiating Crohn’s disease (CD) from other etiologies. **Methods:** This retrospective study included 258 patients who underwent DBE for suspected small-bowel inflammatory lesions at Dokuz Eylül University Hospital (2010–2024). Patients were categorized into the CD and non-CD groups. The clinical, radiological, and endoscopic findings were also analyzed. Statistical comparisons were performed to assess the differences in presentation and DBE findings between the groups. **Results:** The mean patient age was 48.2 ± 17.3 years. Abdominal pain (47.7%) and diarrhea (31.8%) were the most common symptoms. The DBE findings included ulcers (45.0%), superficial mucosal changes (23.3%), and strictures (9.7%). The ileum was the most commonly affected site (31.7%). CD was diagnosed in 27.5% of the patients, while other etiologies included non-steroidal anti-inflammatory drug-induced (NSAID) enteropathy (12.0%) and malignancies (15.9%). Ulcers were significantly more frequent in patients with CD than in those without (60.3% vs. 39.0%, *p* = 0.002). **Conclusions:** DBE plays a crucial role in diagnosing small-bowel inflammatory lesions, distinguishing CD from other conditions, and guiding clinical management. It remains essential for cases requiring histopathological confirmation, offering superior diagnostic accuracy compared to noninvasive imaging.

## 1. Introduction

Beginning at the pylorus and ending at the ileocecal valve, the small bowel is a hollow tube that is 6–7 m long. Various diseases can affect this organ [1]. The most common small-bowel lesions are ulcers and erosions [2,3]. A broad range of differential diagnoses, such as Crohn’s disease (CD), non-steroidal anti-inflammatory drug (NSAID)-related enteritis, cytomegalovirus infection, non-Hodgkin’s lymphoma, cryptogenic multifocal ulcerative stenosing enteritis, and radiation-induced enteritis, can be associated with intestinal erosions and ulcerations [4,5]. Ulcers are the most common manifestation of small-bowel CD [1,6]. The etiology of isolated small-bowel ulcers has always been a challenge. The long and twisted shape of the small bowel makes it difficult to endoscopically investigate and treat ulcerations and erosions of the small bowel. In addition, owing to the wide range of potential diagnoses, the workup for small-bowel strictures may be difficult [7].

The detection of small intestinal mucosal lesions using methods like computed tomography (CT) scanning, magnetic resonance imaging (MRI), and contrast-enhanced abdominal ultrasonography is inadequate, despite significant progress in recent years [8]. Capsule endoscopy (CE) is better than these other techniques in terms of diagnosis, because it involves the thorough examination of the mucosa of the small intestine. CE is a sensitive and noninvasive method for locating intestinal lesions that can reveal mucosal lesions, especially superficial lesions. However, a disadvantage of CE is that biopsies cannot be obtained using this approach [4,9]. Double-balloon enteroscopy (DBE) is a new method used for diagnosing various small-bowel diseases, such as small-bowel tumors, vascular lesions, and inflammatory lesions [10,11,12]. DBE utilizes latex balloons on both the endoscope and the overtube, which can be inflated or deflated using an air-controlled pump. The procedure begins with both balloons deflated, and in the oral route, the endoscope advances to the second part of the duodenum, while the overtube remains behind. The endoscope’s balloon is then inflated, followed by the overtube being advanced distally and its balloon inflated. Through a series of sequential inflation and deflation maneuvers, the endoscope slides deeper into the small bowel, while aligning bowel loops onto the overtube, allowing for deeper insertion. The same push-and-pull technique is used in the anal route, with additional gentle pulling maneuvers to intubate the ileocecal valve and reach the ileum [13]. DBE has the advantage of enabling therapeutic interventions and lesion biopsy in addition to direct imaging of the small-bowel mucosa [10,14]. DBE can be employed in rare situations where pathological tissue investigation is necessary to diagnose suspected malignancy or ambiguous small intestinal disease. Single-balloon enteroscopy, introduced in 2008, offers a simpler and more user-friendly approach to small-bowel endoscopy. However, single-balloon enteroscopy may be less effective than DBE in terms of insertion depth and achieving complete visualization of the small bowel [15].

CD is a chronic, inflammatory condition of the gastrointestinal tract. CD can affect any part of the gastrointestinal tract, from the mouth to the anus. Isolated small-bowel involvement in CD is reported to occur in nearly 30% of patients [16,17]. DBE enables the detection of early mucosal changes that are indicative of CD, including minor erosions, aphthous ulcers, and early inflammatory lesions. Accessing the small bowel using conventional endoscopy is relatively challenging, and the terminal ileum cannot always be successfully navigated using this method. As a result, DBE is often preferred for diagnosing CD that involves the small bowel [18].

The various etiologies of ulceration complicate diagnosis. The aim of this study was to evaluate the usefulness and clinical impact of DBE in patients with inflammatory lesions necessitating endoscopy of the small bowel.

## 2. Methods

### 2.1. Data Collection and Study Design

This retrospective study included patients who underwent DBE for the diagnosis of inflammatory lesions in the small bowel at Dokuz Eylul University between January 2010 and March 2024. The study was conducted in accordance with the Declaration of Helsinki and was approved by the Institutional Review Board of the Dokuz Eylul University Faculty of Medicine Non-Interventional Research Ethics Committee, with decision number 2024/16-35, on 8 May 2024. Patients who underwent DBE due to clinical and radiological findings suggestive of small-bowel inflammation were included in the study.

Indications for DBE included chronic diarrhea, abdominal pain, abdominal masses, perianal lesions, and systemic symptoms such as weight loss, fever, and anemia. Patients underwent esophagogastroduodenoscopy (EGD), colonoscopy, CT, and additional imaging modalities such as MR enterography (MRE) or CE. The oral and anal routes were selected based on the location of the suspected lesion, primarily determined via clinical examinations and previous findings from imaging studies. The DBE approach (oral or anal) was predominantly guided by radiological methods rather than CE, as CE has not been widely used in the past as a primary tool for determining suspected lesion locations in our hospital. DBE was concluded when inflammatory lesions, such as characteristic ulcers or stenosis, were visualized and thoroughly evaluated. The presence, extent, and severity of these lesions were confirmed via DBE, allowing for appropriate diagnostic or therapeutic interventions to be planned based on these findings.

**Figure 1:**
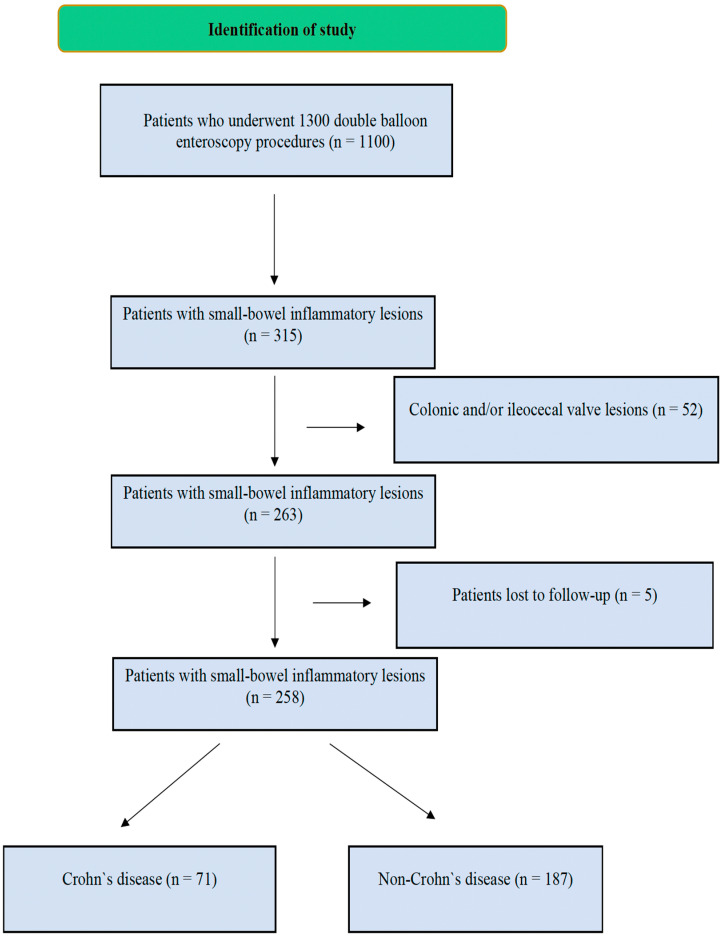
Flowchart of the study.

Patient demographic data (age and gender), clinical symptoms, radiological imaging results, and endoscopic findings during DBE were retrospectively collected from medical records. The type (e.g., ulcers, erosions, or strictures), localization, and size of the lesions were documented in detail. All patients underwent EGD and colonoscopy within the six months prior to DBE examination. Initially, 315 patients with small-bowel inflammatory lesions between the duodenum and ileum identified via DBE were included in the study. Patients with colonic and/or ileocecal valve lesions were subsequently excluded (n = 52). A diagnosis was confirmed via DBE pathological biopsy or postoperative pathology in some patients (n = 57); if the pathology was inconclusive, diagnoses were based on international diagnostic criteria and treatment outcomes. All patients were followed for at least 12 months. After excluding 5 patients lost to follow-up, we enrolled 258 eligible patients for analysis, and they were categorized into 2 groups—Crohn’s disease and non-Crohn’s disease—according to their final diagnosis (Figure 1). We excluded patients aged <18 years.

In our study, all DBE procedures were performed by an endoscopist with 15 years of enteroscopy experience, who had conducted more than 1000 DBE procedures, and were assisted by at least one accompanying doctor using the therapeutic Fujinon DBE System (Fujinon Inc., Saitama, Japan). This system features a 200 cm working length, a soft overtube measuring 140 cm, and a 2.8 mm working channel. The charging and discharging of the two balloons were automatically controlled via an air pump, maintaining a pressure of 6.0–6.5 kPa during discharge and 7.0–7.5 kPa during charging. The procedure was carried out via either the oral or anal route, depending on the suspected lesion location. Biopsies were taken when necessary, and histopathological evaluation was performed. The push-and-pull technique was used, with CO_2_ insufflation or water-assisted methods employed based on the preference of the endoscopist. Oral DBE was performed after a 6 h fasting period, whereas anal DBE required a standard colonic lavage using 2–4 L of a polyethylene glycol–electrolyte solution on the day prior to the procedure. Sedation was administered by an anesthesiologist, with either conscious or deep sedation, while patients were continuously monitored.

The principle of the DBE technique, along with the method used to measure insertion depth, has been previously described in detail in the literature [19,20]. The small-bowel segment was estimated based on both insertion depth and guidance from previous studies. Additionally, if a lesion location was confirmed through other imaging tests, the insertion depth of the scope was adjusted accordingly, using the identified location as a reference. A shortening technique was utilized to achieve the effective deep insertion of the scope without unwanted looping, with fluoroscopy guidance employed in a few procedures (7%). Furthermore, the distinction between the jejunum and ileum during DBE was determined based on the depth of small-bowel insertion, the size of the enteric cavity, and the morphological characteristics of mucosal folds and villi, ensuring the accurate localization of lesions within the small intestine.

An inflammatory lesion (including erythema, erosions, and ulcers) finding was defined as the presence of any significant positive endoscopic finding consistent with the patient’s clinical or radiological presentation. Patients whose final diagnosis could not be determined were classified as having nonspecific small-bowel ulcers. The final diagnosis was defined as a diagnosis that was ultimately decided in consideration of other test results, clinical features, and histopathological results. The diagnosis of CD was confirmed based on clinical symptoms, along with a combination of endoscopic, histological, radiological, and/or biochemical findings [21]. Among the underlying causes of inflammatory lesions, celiac disease is a well-known condition that can manifest with various endoscopic findings. Although atrophic findings are typically expected in DBE, the patients in this study were referred to our hospital for refractory celiac disease and predominantly presented with complications such as erosions and ulcerative jejunitis. In some cases, inflammatory lesions were associated with more severe pathological conditions, including lymphoproliferative diseases and malignancies. Lymphoproliferative diseases specifically referred to lymphomas, including intestinal T-cell and B-cell lymphomas. Malignancies included primary adenocarcinomas, metastatic cancers, and neuroendocrine tumors.

### 2.2. Statistical Analysis

All analyses were performed using the Statistical Package for Social Sciences (SPSS) 24.0. The Kolmogorov–Smirnov and Shapiro–Wilk tests were used for normality tests. Student’s *t*-test was used to compare parametric continuous variables, with the results presented as mean ± SD. Pearson’s chi-square test and Fisher’s exact test were used for categorical variables, with the results given as n (%). Statistical significance was defined as *p* < 0.05.

## 3. Results

### 3.1. Patient Demographics and Baseline Characteristics 

This study included 258 patients undergoing DBE for examination of inflammatory small-bowel lesions. The cohort averaged 48.2 ± 17.3 years. Duodenal involvement was more common in elderly patients (57.1 ± 16.8 years), while panenteritis patients were younger (42.0 ± 15.6 years). The proportion of female patients in the total cohort was 38.8%, with no statistically significant difference between the localization groups. Among the different sites of involvement, female predominance was slightly higher in jejunal lesions (42.9%), while ileal and panenteric involvement showed a more balanced sex distribution (36.6% and 37.8%, respectively). The average duration of the DBE procedures was 50 min, with a range of 20 to 120 min. In our cohort, no major complications, including acute pancreatitis, were observed.

### 3.2. Clinical Presentations and Symptoms

Patients presented with a variety of gastrointestinal and systemic symptoms. The most common presenting symptom was abdominal pain (47.7%), followed by diarrhea (31.8%), anemia (12.0%), and weight loss (14.3%). Other symptoms included nausea (5.4%), steatorrhea (6.6%), and gastrointestinal bleeding (17.1%). Fever was uncommon and was reported in only 1.9% of cases. When stratified by lesion location, abdominal pain was most common in the jejunal (55.6%) and panenteric (51.2%) regions, whereas diarrhea was frequently observed in patients with ileal (37.8%) and panenteric (41.5%) diseases. Gastrointestinal bleeding was noted in 17.1% of cases, with ileal involvement accounting for the highest proportion (19.5%).

### 3.3. Comorbidities and Medication Use

The study population had a diverse range of comorbid conditions, with hypertension (25.6%) being the most prevalent, followed by diabetes mellitus (13.2%), malignancy (11.6%), and dyslipidemia (5.0%). Notably, patients with duodenal involvement had the highest rates of hypertension (32.3%) and coronary artery disease (16.1%). Autoimmune-related disorders, including connective tissue diseases, were more frequently observed in panenteric disease (8.5%). Non-steroidal anti-inflammatory drugs were used by 25.2% of patients, and glucocorticosteroids were prescribed in 2.3% of cases. Anticoagulants (NOACs, warfarin) were infrequent (<2%), whereas selective serotonin reuptake inhibitors were used in 2.7% of patients.

### 3.4. Radiological and Endoscopic Findings

Among the 258 lesions identified, radiological evaluation was performed in 82.6% of cases. Small-bowel wall thickening was the most common radiological finding (31.4%), followed by mass-like lesions (12.0%), lymphadenopathy (2.3%), and subileus/ileus (6.2%). CT scans showed that duodenal involvement was associated with normal imaging findings in 29.0% of cases, whereas jejunal and ileal lesions had higher rates of small-bowel thickening (34.9% and 28.0%, respectively). Notably, mass-like lesions were identified in 8.5% of the ileal cases and 2.4% of the panenteric cases. The clinical characteristics of the patients are summarized in Table 1.

DBE revealed ulcerative lesions in 45.0% of cases, while superficial mucosal changes (23.3%) and edema (22.1%) were also frequently observed. Stenotic lesions were detected in 9.7% of cases, with the highest prevalence observed in ileal involvement (8.5%). Among patients with Crohn’s disease, ulcers were the predominant finding (60.3%), whereas patients with non-CD inflammatory conditions had a lower ulcer prevalence (39.0%) (*p* = 0.002). Superficial changes were observed in 26.7% of non-Crohn’s disease patients compared to 14.1% of CD patients (*p* = 0.032).

### 3.5. Comparison of Crohn’s Disease and Other Diagnoses

Patients diagnosed with CD were significantly younger (40.9 ± 14.7 years vs. 50.9 ± 17.5 years, *p* < 0.001) compared to those diagnosed with other inflammatory conditions. CD patients also had a significantly higher prevalence of abdominal pain (69.0%) and diarrhea (52.1%) compared to non-Crohn’s patients (39.6% and 24.1%, respectively, both *p* < 0.001). The DBE findings revealed a higher prevalence of ulcerative lesions in Crohn’s disease (60.3% vs. 39.0%, *p* = 0.002), while stenosis and superficial changes were more evenly distributed between Crohn’s and non-Crohn’s groups. The characteristics of Crohn’s and non-Crohn’s diseases are summarized in Table 2.

### 3.6. Histopathological and Final Diagnoses

**Figure 2:**
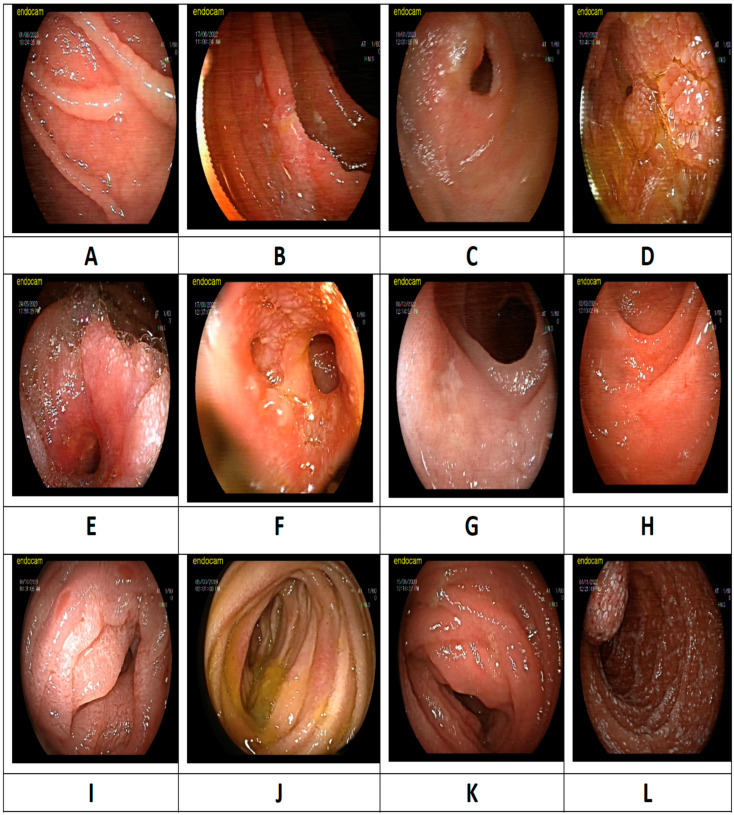
Endoscopic images of small-bowel inflammatory lesions: (**A**) celiac disease, scalloping of jejunal fold; (**B**) celiac disease, ulcerative jejunitis; (**C**) Crohn’s disease, ileal stenosis; (**D**) Crohn’s disease, jejunal stenosis; (**E**) adenocarcinoma, jejunum; (**F**) lymphoma, jejunum; (**G**) non-steroidal anti-inflammatory drug-induced enteropathy, ileum; (**H**) graft-versus-host disease, jejunum; (**I**) autoimmune enteropathy, jejunum; (**J**) eosinophilic enteritis, jejunum; (**K**) amyloidosis, jejunum; and (**L**) lymphangiectasia, jejunum.

Histopathological confirmation was achieved in 57 of the cases through a DBE biopsy or surgical pathology, allowing for more precise diagnosis of small-bowel inflammatory lesions. Table 3 presents the final diagnoses of 258 patients. The most common diagnosis was Crohn’s disease, accounting for 27.5% (n = 71) of cases, followed by malignancy (15.9%, n = 41) and non-specific findings (14.7%, n = 38). Other frequently observed conditions included lesions associated with NSAID use (n = 31, 12.0%) and celiac disease (n = 24, 9.3%). The less common diagnoses included lymphangiectasia (3.5%, n = 9), lymphoproliferative diseases (3.1%, n = 8), eosinophilic enteritis (3.1%, n = 8), infections (2.3%, n = 6), vasculitis (1.9%, n = 5), autoimmune enteropathy (1.9%, n = 5), radiation-induced enteropathy (1.6%, n = 4), amyloidosis (1.2%, n = 3), and ischemic enteropathy (1.2%, n = 3). Rare conditions, such as Behçet’s disease (0.4%, n = 1) and graft-versus-host disease (0.4%, n = 1), were also identified. Endoscopic images of small-bowel inflammatory lesions are shown in Figure 2.

## 4. Discussion

Diagnosing and treating small-bowel inflammatory diseases can be extremely difficult due to the complex structure of the small intestine and the limitations of traditional diagnostic techniques. With its direct visibility, targeted biopsies, and therapeutic interventions, double-balloon enteroscopy has become a crucial tool in improving diagnostic precision and influencing clinical judgment. In this study, we assessed the diagnostic accuracy and clinical impact of DBE, emphasizing its role in refining preliminary diagnoses amd distinguishing Crohn’s disease from other inflammatory conditions. Our findings reinforce DBE’s indispensable role in small-bowel disease evaluation, aligning with previous research demonstrating its high diagnostic yield and ability to alter clinical management in a significant proportion of patients [22].

Abdominal pain (47.7%) was the most common presenting symptom in our cohort, consistent with previous reports that investigated CD [5,9]. Our patient population consisted of patients with suspected inflammatory lesions; therefore, abdominal pain was the most common symptom. In contrast, gastrointestinal bleeding has been reported more frequently in DBE series investigating all small-bowel diseases [22,23,24]. Abdominal pain was more frequent in jejunal (55.6%) and panenteric (51.2%) disease. Furthermore, diarrhea was more frequent in ileal (37.8%) and panenteric (41.5%) disease, supporting the established pathophysiological role of small-bowel inflammation in malabsorption syndromes. Gastrointestinal bleeding was noted in 17.1% of cases, predominantly in ileal lesions (19.5%), and ileal involvement was strongly associated with occult or overt bleeding in inflammatory enteropathies.

Inflammatory lesions are among the most common lesions in the small bowel [15,22]. The distribution of inflammatory lesions was consistent with previous epidemiological data; the most frequent etiology of inflammatory lesions in our cohort was Crohn’s disease (27.5%) [22]. In a study of 18 patients with suspected isolated small-bowel Crohn’s disease, Crohn’s disease was confirmed in 14 patients (78%). Among the remaining four patients, various inflammatory lesions were considered, including duodenal carcinoma in one patient [9]. This difference may be due to the small sample size of the patient population. Similarly to our study, the prevalence of a new CD diagnosis in a total of 100 patients was 38% [25].

The most commonly affected site was the ileum (31.7%), followed by the jejunum (23.0%) and duodenum (13.1%), supporting prior findings on the ileum’s predominant involvement in Crohn’s disease [26] and NSAID-induced enteropathy [22]. Additionally, our study revealed that patients with duodenal lesions were significantly older (mean age: 57.1 years), whereas panenteric disease was more frequently observed in younger patients (mean age: 42.0 years). This pattern is in line with research showing age-related differences in small-bowel inflammation, where NSAID-related enteropathy is more prevalent in older individuals, while immune-mediated disorders like Crohn’s disease and Behçet’s disease are more common in younger populations.

CT enterography, MR enterography, and capsule endoscopy are widely utilized for small-bowel evaluation. However, they have notable limitations in obtaining histological samples and performing therapeutic interventions, making DBE a gold standard method for cases requiring mucosal assessment. While CT enterography and MR enterography are useful for assessing transmural inflammation and strictures, their sensitivity in detecting superficial mucosal lesions remains limited [27]. In suspected CD patients, the diagnostic yield was 30%, while in individuals with established CD, it was roughly 60%. The yield increased if the lesion’s location had been confirmed by earlier tests [24,28]. In one study, the diagnostic yields of DBE were higher than those of CT enterography for inflammatory diseases [27]. The diagnosis of small-bowel CD can be supported pathologically via DBE [18]. Instead, alternative diagnoses such as NSAID-induced enteropathy, eosinophilic enteritis, autoimmune enteropathy, and Behçet’s disease were established following DBE assessment, supporting previous research suggesting that many small-bowel inflammatory conditions mimic Crohn’s disease, but have distinct pathophysiological mechanisms and treatment requirements [5]. Similarly, CE is a highly sensitive modality for detecting small-bowel mucosal lesions, but it lacks biopsy capability and is limited by retention risk in patients with strictures [29]. Our study corroborates findings from previous research suggesting that DBE remains the most effective technique for both diagnosing and characterizing inflammatory small-bowel lesions, particularly in cases where histopathologic confirmation is required [5]. In a previous study, small-bowel ulcers were identified via CE in a significant percentage of patients with suspected Crohn’s disease, but DBE led to a higher percentage of definitive diagnoses due to its ability to obtain biopsies and rule out alternative etiologies [30]. There is no established set of endoscopic criteria for diagnosing Crohn’s disease. In this study, the observed endoscopic findings ranged from classic features to more subtle or early signs, including aphthous and small ulcers. The DBE findings indicated that ulcerative lesions were significantly more prevalent in the Crohn’s disease group (60.3% vs. 39.0%, *p* = 0.002), whereas the distribution of stenosis and superficial changes was comparable between the Crohn’s and non-Crohn’s groups. DBE remains the gold standard for evaluating small-bowel inflammatory diseases, given its unique ability to provide direct mucosal visualization, obtain histological samples, and perform therapeutic interventions. While noninvasive imaging techniques play a crucial role in initial assessment, DBE is indispensable for establishing a definitive diagnosis and guiding appropriate treatment strategies.

This study had some limitations. As this was a retrospective, single-center study, the results may not be generalizable to all populations. However, the relatively large sample size enhanced the reliability of the findings. While double-balloon enteroscopy allows for direct mucosal visualization and biopsy, some deeper or less accessible small-bowel regions may not be fully evaluated.

## 5. Conclusions

Double-balloon enteroscopy plays a crucial role in the evaluation and management of inflammatory lesions of the small bowel. In this study, DBE provided direct visualization, biopsy capability, and therapeutic interventions, contributing to accurate diagnosis and improved clinical decision making. Crohn’s disease was the most common etiology, but a wide spectrum of inflammatory conditions, including NSAID-related enteropathy and malignancies, was also identified. Our findings highlight the importance of DBE in differentiating small-bowel pathologies, particularly in cases where noninvasive imaging methods are inconclusive. Although DBE remains a valuable tool, combining it with other diagnostic modalities, such as video capsule endoscopy and cross-sectional imaging, may further enhance diagnostic accuracy. Future studies with larger multicenter cohorts and longer follow-up periods will be beneficial in refining the diagnostic and therapeutic strategies for small-bowel inflammatory diseases.

## Figures and Tables

**Table 1:** Comparison of features between duodenal, jejuneal, ileal, and all sites.

	Duodenum (n = 31)	Jejunum (n = 63)	Ileum (n = 82)	Panenteritis (n = 82)	Total (n = 258)
Age, mean ± SD	57.1 ± 16.8	52.7 ± 17.2	46.6 ± 17.5	42.0 ± 15.6	48.2 ± 17.3
Female sex, *n* (%)	12 (38.7)	27 (42.9)	30 (36.6)	31 (37.8)	100 (38.8)
Complaint at admission, *n* (%)					
Abdominal pain	10 (32.3)	35 (55.6)	36 (43.9)	42 (51.2)	123 (47.7)
Diarrhea	5 (16.1)	12 (19.0)	31 (37.8)	34 (41.5)	82 (31.8)
Nausea	5 (16.1)	2 (3.2)	1 (1.2)	6 (7.3)	14 (5.4)
Bleeding	8 (25.8)	10 (15.9)	16 (19.5)	10 (12.2)	44 (17.1)
Steatorrhea	2 (6.5)	3 (4.8)	3 (3.7)	9 (11.0)	17 (6.6)
Weight loss	6 (19.4)	10 (19.4)	8 (9.8)	13 (15.9)	37 (14.3)
Anemia	5 (16.1)	10 (15.9)	9 (11.0)	7 (8.5)	31 (12.0)
Ileus/subileus	0 (0)	3 (4.8)	15 (18.3)	9 (11.0)	27 (10.5)
Fever	1 (3.2)	1 (1.6)	0 (0)	3 (3.7)	5 (1.9)
Comorbidity, *n* (%)					
Hypertension	10 (32.3)	25 (39.7)	17 (20.7)	14 (17.1)	66 (25.6)
DM	4 (12.9)	12 (19)	11 (13.4)	7 (8.5)	34 (13.2)
Dyslipidemia	2 (6.5)	4 (6.3)	6 (7.3)	1 (1.2)	13 (5.0)
CAD	5 (16.1)	5 (7.9)	6 (7.3)	3 (3.7)	19 (7.4)
Heart failure	0 (0)	2 (3.2)	3 (3.7)	0 (0)	5 (1.9)
Afib	2 (6.5)	4 (6.3)	1 (1.2)	2 (2.4)	9 (3.5)
COPD	1 (3.2)	3 (4.8)	5 (6.1)	1 (1.2)	10 (3.9)
CKD	1 (3.2)	1 (1.6)	2 (2.4)	0 (0)	4 (1.6)
Immunodeficiency	0 (0)	0 (0)	1 (1.2)	4 (4.9)	5 (1.9)
Hypothyroidism	3 (9.7)	2 (3.2)	2 (2.4)	1 (1.2)	8 (3.1)
Malignancy	6 (19.4)	11 (17.5)	7 (8.5)	6 (7.3)	30 (11.6)
Liver cirrhosis	0 (0)	1 (1.6)	2 (2.4)	1 (1.2)	4 (1.6)
CTD	0 (0)	3 (4.8)	5 (6.1)	7 (8.5)	15 (5.8)
Medication, *n* (%)					
NSAIDs	5 (16.1)	13 (20.6)	25 (28.0)	24 (29.3)	65 (25.2)
ASA	6 (19.4)	9 (14.3)	11 (13.4)	4 (4.9)	30 (11.6)
SSRIs	1 (3.2)	2 (3.2)	3 (3.3)	1 (1.2)	7 (2.7)
NOACs	1 (3.2)	3 (4.8)	1 (1.2)	0 (0)	5 (1.9)
Warfarin	0 (0)	1 (1.6)	1 (1.2)	2 (2.4)	4 (1.6)
Glucocorticosteroids	0 (0)	1 (1.6)	3 (3.7)	2 (2.4)	6 (2.3)
Metformin	1 (3.2)	6 (9.5)	0 (0)	3 (3.7)	10 (3.9)
History of GIS surgery, *n* (%)	2 (6.5)	1 (1.6)	8 (9.8)	1 (1.2)	12 (4.6)
CT findings, *n* (%)					
Not performed	7 (22.6)	7 (11.1)	15 (18.3)	16 (19.5)	45 (17.4)
Normal	9 (29.0)	14 (22.2)	27 (32.9)	28 (34.1)	78 (30.2)
Wall thickness	6 (19.4)	22 (34.9)	23 (28.0)	30 (36.6)	81 (31.4)
Mass appearance	5 (16.1)	17 (27.0)	7 (8.5)	2 (2.4)	31 (12.0)
Bleeding	0 (0)	0 (0)	0 (0)	1 (1.2)	1 (0.4)
Lymphadenopathy	0 (0)	2 (3.2)	2 (2.4)	2 (2.4)	6 (2.3)
Ileus/subileus	4 (12.9)	1 (1.6)	8 (9.8)	3 (3.7)	16 (6.2)
Double-balloon enteroscopy findings, *n* (%)					
Superficial changes	4 (12.9)	14 (22.2)	21 (25.6)	21 (25.6)	60 (23.3)
Edema	7 (22.6)	16 (25.4)	7 (8.5)	27 (32.9)	57 (22.1)
Ulcer	17 (54.8)	25 (39.7)	47 (57.3)	27 (32.9)	116 (45.0)
Stenosis	3 (9.7)	8 (12.7)	7 (8.5)	7 (8.5)	25 (9.7)

Abbreviations: DM, diabetes mellitus; Afib, atrial fibrillation; COPD, chronic obstructive pulmonary disease; CKD, chronic kidney disease; CTD, connective tissue disorder; NSAIDs, non-steroidal anti-inflammatory drugs; ASA, acetylsalicylic acid; SSRIs, selective serotonin reuptake inhibitors; NOACs, new-generation oral anticoagulants; GIS, gastrointestinal system; and CT; computerized tomography.

**Table 2:** Comparison of features between Crohn’s disease and non-Crohn’s disease.

	Crohn’s Disease (n = 71)	Non-Crohn’s Disease (n = 187)	*p*-Value
Age, mean ± SD	40.9 ± 14.7	50.9 ± 17.5	<0.001
Female sex, *n* (%)	26 (36.6)	74 (39.6)	0.664
Complaint at admission, *n* (%)			
Abdominal pain	49 (69.0)	74 (39.6)	<0.001
Diarrhea	37 (52.1)	45 (24.1)	<0.001
Nausea	2 (2.8)	12 (6.4)	0.362
Bleeding	2 (2.8)	42 (22.5)	<0.001
Steatorrhea	4 (5.6)	13 (7.0)	0.703
Weight loss	11 (15.5)	26 (13.9)	0.745
Anemia	3 (4.2)	28 (15.0)	0.018
Ileus/subileus	13 (18.3)	14 (7.5)	0.011
Fever	0 (0)	5 (2.7)	0.327 *
Comorbidities, *n* (%)			
Hypertension	9 (12.7)	57 (30.5)	0.003
DM	4 (5.6)	30 (16.0)	0.027
Dyslipidemia	2 (2.8)	11 (5.9)	0.524
CAD	2 (2.8)	17 (9.1)	0.085
Heart failure	0 (0)	5 (2.7)	0.327 *
Afib	0 (0)	9 (4.8)	0.067 *
COPD	2 (2.8)	8 (4.3)	0.732
CKD	0 (0)	4 (2.1)	0.578 *
Immunodeficiency	0 (0)	5 (2.7)	0.327 *
Hypothyroidism	1 (1.4)	7 (3.7)	0.452 *
Malignancy	4 (5.6)	26 (13.9)	0.064
Liver cirrhosis	1 (1.4)	3 (1.6)	0.909 *
CTD	8 (11.3)	7 (3.7)	0.021
Medications, *n* (%)			
NSAIDs	14 (19.7)	51 (27.3)	0.212
ASA	8 (11.3)	22 (11.8)	0.911
SSRIs	1 (1.4)	6 (3.2)	0.677 *
NOACs	0 (0)	5 (2.7)	0.327 *
Warfarin	0 (0)	4 (2.1)	0.578 *
Glucocorticosteroids	1 (1.4)	5 (2.7)	0.889 *
Metformin	2 (2.8)	8 (4.3)	0.732
History of GIS surgery, *n* (%)	3 (4.2)	9 (4.8)	0.841
Intestinal involvement, *n* (%)			N/A
Duodenum (only)	3 (4.2)	28 (15.0)	
Jejunum (only)	5 (7.0)	58 (31.0)	
Ileum (only)	39 (54.9)	43 (23.0)	
Duodenum and jejunum	2 (2.8)	26 (13.9)	
Duodenum and ileum	2 (2.8)	6 (3.2)	
Jejunum and ileum	13 (18.3)	18 (9.6)	
Panenteritis	7 (9.9)	8 (4.3)	
CT findings, *n* (%)			N/A
Not performed	9 (12.7)	36 (19.3)	
Normal	19 (26.8)	59 (31.6)	
Wall thickness	29 (40.8)	52 (27.8)	
Mass appearance	3 (4.2)	28 (15.0)	
Bleeding	0 (0)	1 (0.5)	
Lymphadenopathy	3 (4.2)	3 (1.6)	
Ileus/subileus	8 (11.4)	8 (4.3)	
Double-balloon enteroscopy findings, *n* (%)			
Superficial changes	10 (14.1)	50 (26.7)	0.032
Edema	11 (15.5)	46 (24.6)	0.115
Ulcer	43 (60.3)	73 (39.0)	0.002
Stenosis	7 (9.9)	18 (9.6)	0.955

Abbreviations: DM, diabetes mellitus; Afib, atrial fibrillation; COPD, chronic obstructive pulmonary disease; CKD, chronic kidney disease; CTD, connective tissue disorder; NSAIDs, non-steroidal anti-inflammatory drugs; ASA, acetylsalicylic acid; SSRIs, selective serotonin reuptake inhibitors; NOACs, new-generation oral anticoagulants; GIS, gastrointestinal system; CT; computerized tomography; N/A, not applicable; and * Fisher’s exact test was used.

**Table 3:** Final diagnosis features of the patients.

Final Diagnosis	n = 258	Percentage (%)
Crohn’s disease	71	27.5%
Non-steroidal anti-inflammatory drug-induced enteropathy	31	12.0%
Celiac	24	9.3%
Radiation enteritis	4	1.6%
Infection	6	2.3%
Ischemia	3	1.2%
Autoimmune enteropathy	5	1.9%
Behcet’s disease	1	0.4%
Lymphoma	8	3.1%
Malignancy ^a^	41	15.9%
Eosinophilic enteritis	8	3.1%
Vasculitis	5	1.9%
Graft-versus-host disease	1	0.4%
Lymphangiectasia	9	3.5%
Amyloidosis	3	1.2%
Non-specific	38	14.7%

^a^: Includes adenocarcinomas, metastatic cancers, and neuroendocrine tumors.

## Data Availability

The datasets used and/or analyzed in this study are available upon reasonable request from the corresponding author.
